# Influence of Jasmine Tea Scenting Cycles on Volatile Transfer and Sensory Quality in Light Milk Tea

**DOI:** 10.3390/foods15193484

**Published:** 2026-09-29

**Authors:** Junying Fan, Jiangli Sun, Wei Li, Tianyuan Cheng, Pushpendra Koli, Rui Wang, Yingjie Ma, Litao Sun

**Affiliations:** 1 Tea Research Institute, Shandong Academy of Agricultural Sciences, Jinan 250100, China; fanjunying@mxbc.com (J.F.); m15864388277@163.com (T.C.); 2 D. CO International Food Co., Ltd., Jiaozuo 454850, China; sunjiangli@mxbc.com (J.S.); 15063925982@163.com (W.L.); wangrui01@mxbc.com (R.W.); mayingjie@mxbc.com (Y.M.); 3 Indian Grassland and Fodder Research Institute, Indian Council of Agricultural Research (ICAR), Jhansi 284003, UP, India; kolipushpendra@gmail.com; 4 College of Science, Health, Engineering and Education, Murdoch University, Perth, WA 6150, Australia

**Keywords:** light milk tea, matrix effects, sensory analysis, scenting, beverage

## Abstract

Jasmine tea, a traditionally scented tea, has increasingly been used as a base ingredient for emerging tea beverages such as light milk tea. However, how variations in scenting intensity affect aroma characteristics in complex milk matrices remains unclear. This study investigated the effects of scenting cycles (1 to 3 rounds) on the sensory characteristics and volatile profiles of jasmine teas and their corresponding light milk teas. Sensory characteristics were evaluated by a trained panel using descriptive sensory analysis, while volatile profiles were characterized using HS-SPME-GC-MS-based flavor omics. Results showed that increasing scenting cycles significantly enhanced aroma intensity, freshness, and persistence in pure tea infusions. However, sensory differences between two- and three-scented teas largely converged after incorporation into the milk matrix. Volatile profiling revealed that the milk system markedly attenuated compositional differences originating from the tea base. Transfer factor analysis identified esters and ketones as the most stably retained compound classes, with methyl anthranilate, linalool, and benzyl alcohol defined as core transfer compounds. Importantly, the conventional jasmine tea flavor index was effective for pure jasmine tea evaluation but failed to capture aroma quality in milk tea, highlighting the necessity for a matrix-specific evaluation framework. This study provides an integrated framework for flavor assessment that bridges traditional tea processing and modern beverage development.

## 1. Introduction

Tea is one of the most widely consumed non-alcoholic beverages worldwide [1]. As a representative of traditional scented teas in China, jasmine tea exhibits a characteristic flavor profile derived from the harmonious integration of tea leaves and the jasmine flowers (*Jasminum sambac* (L.) Aiton) fragrance [2]. It occupies a prominent position in the global tea market, serving not only as a traditional daily beverage but also as a crucial base for new-style tea drinks [3,4].

The production of jasmine tea can be traced back to the Song Dynasty. After centuries of refinement, a unique processing system centered on “scenting” has been established. This process endows tea with floral and fresh flavor attributes through the adsorption and bonding of aroma compounds released from fresh jasmine flowers onto the tea base [5]. Studies have confirmed that jasmine tea not only possesses a distinctive flavor but also is rich in bioactive substances such as polyphenols, terpenoids, and alkaloids, exhibiting health benefits including antioxidant activity, mood regulation, and antidepressant effects [6,7]. As the world’s largest producer of jasmine flowers and jasmine tea, China exports its products from core producing regions such as Fuzhou (Fujian Province) and Hengzhou (Guangxi Zhuang Autonomous Region) to overseas markets. In 2023, total export volume reached 6210.55 tons, covering 69 countries and regions, highlighting its significant industrial value [8].

The core flavor of jasmine tea originates from the adsorption and retention of volatile compounds from fresh jasmine flowers by the tea base, with the scenting process being a key step regulating aroma formation. Traditional scenting involves tea base pretreatment, fresh flower preservation, tea-flower mixing and piling, aeration for heat dissipation, flower separation, and drying [9]. The number of scenting cycles is a core parameter affecting flavor development. Repeated scenting gradually accumulates aroma compounds, directly influencing the concentration, purity, and persistence of the aroma in the tea base, while also significantly increasing production costs [10]. In traditional production, jasmine tea typically undergoes 1 to 7 scenting cycles, with high-grade products requiring 5 to 7 cycles. The/adsorption capacity of the tea base for jasmine aroma compounds significantly improves with increasing scenting cycles [11]. Pang et al. [12] analyzed jasmine tea with different scenting gradients using HS-SPME-GC-MS and HS-GC-IMS techniques and found that high-grade tea subjected to five scenting cycles maintained stable aroma release even after the fourth infusion. Furthermore, Shen et al. [13] identified key odorants such as β-ionone, linalool, indole, and methyl anthranilate as major contributors to the floral aroma of jasmine tea. From the perspective of the adsorption mechanism, the process also alters the physical structure of the tea base; the porous structure can enrich aroma molecules through condensation, while macromolecules like polyphenols may bind to aroma components via hydrogen bonds, thereby improving retention [14]. Yang et al. [15] showed that transfer to the tea base is multi-factor dependent, while retention in spent flowers is mainly governed by hydrophobic partitioning. However, current research on gradient scenting mostly focuses on the identification of aroma components in the tea base. For example, Lin et al. [16] proposed the “jasmine tea flavor (JTF) index” which evaluates scenting quality using the ratio of the total peak area of α-farnesene, (Z)-3-hexenyl benzoate, methyl anthranilate, and indole to the peak area of linalool. However, systematic research remains insufficient on flavor differences among tea bases with different scenting gradients and their influences on downstream products such as light milk tea.

The flavor quality of jasmine tea is jointly determined by the type, content, and proportion of volatile aroma compounds. Existing studies have identified a variety of key flavor substances. Esters often constitute the highest proportion among detected volatiles, with components like benzyl phenylacetate, linalool, methyl anthranilate, and indole identified as core contributors to the floral aroma [12]. Chen et al. [17] found in their study on scented tea that benzoic acid, 2-hydroxyethyl ester was the key component imparting a sweet aroma to scented tea, while (Z)-3-hexenyl benzoate and methyl salicylate enhanced the fresh note. In contrast, linalool, methyl anthranilate, and benzyl alcohol were the core substances contributing to the rich floral aroma of jasmine-scented tea. In addition to volatile components, non-volatile substances in the tea base (including polyphenols, caffeine, free amino acids) regulate flavor perception through interactions with aroma compounds [18]. However, most existing studies focus on pure tea systems, and research on the flavor performance of tea bases in composite beverages remains scarce.

In recent years, the freshly made and ready-to-drink (RTD) tea beverage market has experienced rapid expansion. Light milk tea, characterized by a “fresh tea aroma + mild milk aroma” profile, has become a major driver of the new-style tea beverage industry among young consumers. This trend has promoted the extension of jasmine tea from a traditional beverage to a diversified deep-processing field [19,20]. In light milk tea, the addition of dairy products not only changes the physicochemical environment of the system but also may affect the release rate and perception threshold of aroma compounds through interactions between fats/proteins and volatile compounds in tea [21,22]. Currently, there are evident knowledge gaps regarding the application of gradient-scented tea bases in light milk tea. The retention and release patterns of key aroma components from differentially scented tea bases in milk systems are not yet clear. Moreover, the interaction mechanisms of flavor substances in the tea–milk system and the sensory preferences of consumers for light milk tea made from gradient-scented tea bases lack systematic data support. Specifically, the following three shortcomings remain: (i) How do scenting cycles systematically alter the accumulation of key aroma compounds in jasmine tea? (ii) Which volatile compounds exhibit high transfer efficiency and stability from tea to milk tea systems? (iii) Is the traditional JTF index applicable for evaluating aroma quality in composite beverages such as light milk teas? If not, what alternative indicators could be proposed?

To address these gaps, this study investigated jasmine teas produced with one to three scenting cycles and their corresponding light milk teas by integrating sensory evaluation, HS-SPME-GC–MS-based volatilomics, and transfer factor (TF) analysis. This work links traditional tea processing with modern product development, offering practical insights for producing high-quality, cost-effective tea beverages.

## 2. Materials and Methods

### 2.1. Sample Information of Jasmine Tea

The jasmine tea samples were produced in Fuzhou, China, between 5 June and 10 July 2025. The base tea, produced in Zhouning, Ningde City, Fujian Province, was made from *Camellia sinensis* (L.) Kuntze cv. Fudingdabai tea shoots (one bud and two or three leaves) harvested in late May to early June. The tea processing included drum roasting at approximately 300 °C, rolling for 45–55 min, primary drying at 100–110 °C, and secondary drying at 90 °C, followed by refining steps including screening, cutting, re-screening, air separation, color sorting, and stacking. Jasmine flowers (*Jasminum sambac* cv. bifoliatum) purchased from Hengzhou Country, Nanning City, Guangxi Zhuang Autonomous Region, were used as the scenting material. Jasmine flowers were picked as unopened mature buds from 3 to 5 pm on the day of scenting. Jasmine flowers were processed as follows: the flowers were thinly spread to cool, followed by repeated piling and spreading 3–5 times until ≥60% of buds opened; when ≥80% of buds had opened, they were used for scenting. A small amount of Michelia alba flowers (1%, *w*/*w*, about 1 kg of jasmine flowers were mixed with 100 kg of base tea) was added as a traditional base note, according to the Chinese national standard (GB/T 34779-2017) [23]. This level was kept constant across all scented treatments (T1–T3) and was not added to the unscented control (CK). For the first scenting round (5 June 2025), about 60 kg of jasmine flowers were mixed with 100 kg of base tea (CK). The pile was maintained below 45 °C by spreading when necessary; after 2 h, the flowers were removed, and the tea was dried at ∼110 °C to below 6% moisture, yielding sample T1. The second round (19 June 2025) used 40 kg flowers per 100 kg T1 and was dried at 105 °C, producing T2. The third round (10 July 2025) used 30 kg flowers per 100 kg T2 and was dried at 95 °C, resulting in sample T3. Three independent scenting experiments were conducted for each treatment using separate tea batches, and samples obtained from each experiment were treated as independent experimental replicates. All samples were stored at 4 °C for further analysis.

### 2.2. Sample Information of Light Milk Teas

The jasmine tea infusion was prepared by steeping 6 g of samples (CK, T1, T2, T3) in 150 mL of hot water (90–95 °C) for 8 min. The infusion was then filtered into 15 g of ice cubes, stirred, and cooled. Subsequently, light milk teas were prepared using a standardized laboratory-scale formula supplied by the collaborating beverage company: 50 g of milk (formulated dairy ingredient) was combined with 150 g of the prepared tea infusion and 30 g of syrup. Additional ice (170 g) and water (30 g) were added to bring the final volume to 430 mL, and the final temperature was approximately 3–4 °C. The milk (formulated dairy ingredient, 19.70% fat, 2.90% protein, 10.20% carbohydrate, pH 7.2) and syrup (sucrose syrup, pH 4.0, 71.5 °Bx) were provided by D. CO International Food Co., Ltd (Wen Country, Jiaozuo City, Henan Province, China). The light milk tea samples prepared from CK, T1, T2, and T3 were designated as MCK, MT1, MT2, and MT3, respectively. Three biological replicates were prepared independently for each sample, snap-frozen in liquid nitrogen and stored at −80 °C for subsequent analysis. For sensory evaluation, samples were prepared fresh and evaluated within 15 min after preparation.

### 2.3. Sensory Evaluation of Jasmine Teas and Light Milk Teas

The sensory evaluation of jasmine teas and light milk teas was conducted by a trained panel of seven experts, including three males and four females, aged 30–48 years. The seven panelists received more than two days of training, during which attribute definitions, reference samples, and scoring procedures were standardized. Sensory evaluations were conducted in a dedicated sensory evaluation room under controlled lighting, temperature, and odor-free conditions in Tea Research Institute, Shandong Academy of Agricultural Sciences. Samples were presented in a randomized, three-digit coded order under controlled lighting and ambient odor-free conditions. For the jasmine tea base for light milk tea preparation, the sensory evaluation method was adapted from the Chinese national standards (GB/T 23776-2018 and GB/T 22292-2017) [24,25]. Instead of the full set of attributes (appearance, aroma, taste, infusion color, and infused leaves), we focused on aromatic freshness, purity, intensity, persistence, and overall taste. Specifically, 3 g of jasmine tea leaves were infused with 150 mL of freshly boiled water. The first infusion (3 min) was evaluated primarily for the freshness and purity of the jasmine aroma, while the second infusion (5 min) was assessed for aroma concentration, persistence, and the overall taste. The light milk tea samples were prepared according to a standardized formulation and evaluated directly in their final state. The intensity of key sensory attributes including aroma, taste and tea–milk integration was quantified using unstructured line scales aligned with ISO 4121:2003 [26]. Thereinto, tea–milk integration was defined as the perceived balance and harmonious blending between tea aroma/astringency and milk creaminess/sweetness, with no obvious separation or dominance of either phase. Panel-rated overall acceptability was defined as “Acceptability”, including like lightly, like moderately and like extremely. Panelists were instructed to place a vertical mark on the line at the position that best represented their perception. Before formal evaluation, all panelists underwent training using representative samples to standardize attribute definitions and scoring criteria. Mean scores were used for the quantitative description of sample quality.

### 2.4. Determination of Volatile Compounds Using HS-SPME-GC-MS

Frozen samples (jasmine teas and light milk teas) stored at −80 °C were pulverized under liquid nitrogen and homogenized by vortexing (MM400, Retsch, Haan, Germany). Approximately 500 mg of each sample was weighed into a 20 mL headspace vial (Agilent, Palo Alto, CA, USA), followed by the addition of 2 mL of saturated NaCl solution and 20 μL of 10 μg/mL 3-hexanone-2,2,4,4-d4 (CAS: 24588-54-3) internal standard [27]. Volatile compounds were extracted using gas chromatography-mass spectrometry (GC-MS) combined with solid-phase microextraction (SPME). The SPME fiber (120 μm DVB/CWR/PDMS) was exposed to the vial headspace for 15 min, under constant temperature with agitation (5 min) at 80 °C for jasmine teas and 60 °C for light milk teas and then desorbed in the GC injector at 250 °C for 5 min. Separation and identification were carried out using an Agilent 8890-7000D GC-MS system (Agilent Technologies, Santa Clara, CA, USA) equipped with a DB-5MS capillary column (30 m × 0.25 mm × 0.25 μm, Agilent J &W Scientific, Folsom, CA, USA). High-purity helium (≥99.999%) was used as the carrier gas at a constant flow rate of 1.2 mL/min. The injector temperature was set at 250 °C with a solvent delay of 3.5 min. The oven temperature program was as follows: hold at 40 °C for 3.5 min, ramp to 100 °C at 10 °C/min, then to 180 °C at 7° C/min, and finally to 280 °C at 25 °C/min, holding for 5 min. Mass spectrometry detection was performed utilizing an electron impact (EI) ion source at 230 °C, with the quadrupole and transfer line temperature set at 150 °C, and 280 °C. The ionization energy was 70 eV, and data were acquired in the selected ion monitoring (SIM) mode).

### 2.5. Identification and Semi-Quantification of Volatile Compounds

Volatile compounds were identified by matching experimental data against a custom mass spectral database constructed from authentic chemical standards, retention indices (RI), mass spectra from the NIST library and literature reports. For reliable identification and quantification in selected ion monitoring (SIM) mode, the database recorded the expected retention time (RT) along with one quantitative ion and two to three qualifying ions for each compound. During analysis, target ions were monitored within specific time windows corresponding to their elution order. A compound was confirmed when its observed retention time aligned with the reference value and its mass spectrum exhibited the selected ions after background correction [28]. Semi-quantification was performed by integrating the peak areas of the quantitative ions and calibrating using the internal standard to ensure accuracy. For each compound, one quantifier ion and two to three qualifier ions were selected. The relative content of each compound (X_i_, μg/g) in the sample was calculated using the internal standard method (Equation (1)):

X_i_ = (V_s_ × C_s_/M) × (I_i_/I_s_) × 10^−3^ (1)

V_s_ is the volume of the internal standard added (μL), C_s_ is the concentration of the internal standard (μg/mL), M is the mass of the sample (g), I_s_ is the peak area of the internal standard, and I_i_ is the peak area of the target compound.

### 2.6. Data and Statistical Analysis

To evaluate the contribution of each aroma compound to the overall flavor profile, the Relative Odor Activity Value (ROAV) is determined according to Equation (2):

ROAV_i_ = (Ci/OTi) × (Tmax/Cmax) × 100 (2)

where C_i_ is the relative content of the compound (μg/g), OT_i_ is its odor threshold in water (μg/g) and Tmax/Cmax is the maximum value of threshold-to-content ratio among all detected volatile compounds. According to previous studies, ROAV ≥ 1 indicates a significant contribution to the overall aroma [29,30].

The jasmine tea flavor (JTF) index is directly proportional to aroma concentration and freshness [16]. Therefore, the quality of the aroma is evaluated by JTF (Equation (3)).

JTF index = peak area percentage of (Farnesene + 3-Hexenol Benzoate + Indole + 2-Methyl Aminobenzoate)/Linalool(3)

To compare how scenting-related changes in volatile abundance observed in the tea base were reflected in the corresponding milk tea, an operational transfer factor (TF) was calculated as the ratio of the fold change in compound content in the milk tea system to the corresponding fold change in the tea base system according to Equation (4):

TF = (Fold Change in Milk Tea)/(Fold Change in Tea Base)(4)

where fold change is calculated relative to the first scented level (MT3/MT1 vs. T3/T1 and MT2/MT1 vs. T2/T1). A TF > 1.5 indicates enhanced transfer from jasmine tea to the light milk tea (Type E, enhanced aroma); 0.7 < TF < 1.5 indicates stable transfer (Type S: stable aroma); TF < 0.7 indicates depleted transfer (Type D: depleted aroma).

**Figure 1:**
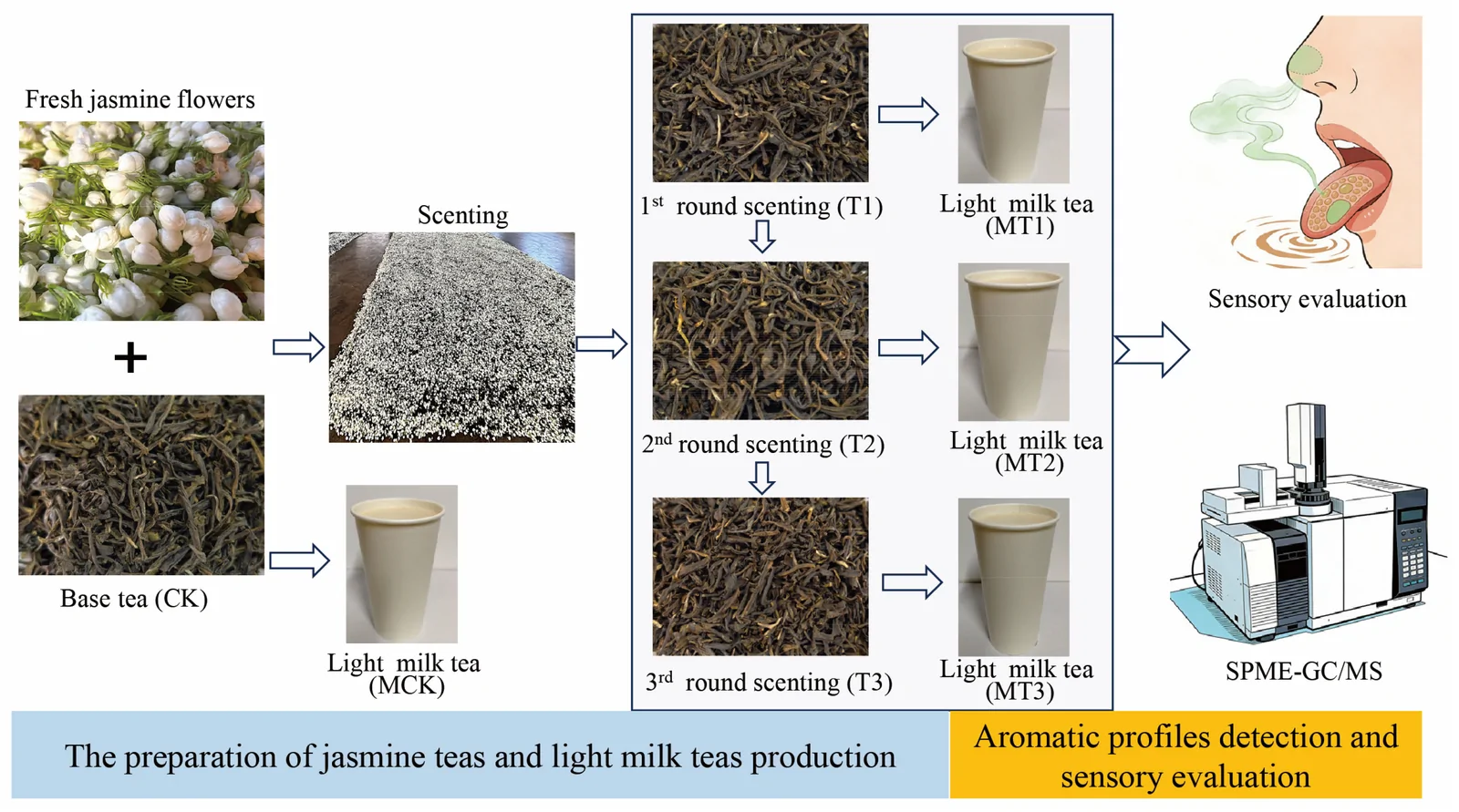
Flow chart of the experiment design.

Initial data organization and calculations were performed using Microsoft Excel 2010. Data visualization, including radar charts, bar charts, and pie charts, was conducted with Origin 2021 (OriginLab Corp., Northampton, MA, USA). Multivariate statistical analysis, including Venn diagrams, heatmaps, principal component analysis (PCA) and orthogonal partial least squares-discriminant analysis (OPLS-DA) were performed using the Metware Cloud, accessed on 20 October 2025, a free online platform for data analysis (https://cloud.metware.cn). Thereinto, the data pre-processing of the relative contents of all identified VOCs for PCA was Z-score and OPLS-DA was log2-transformed and Zero-centered to reduce heteroscedasticity and enhance signal from less abundant compounds. Variable importance in projection (VIP) scores was derived from the OPLS-DA models to identify metabolites contributing most to group separation. Hierarchical clustering heatmaps were was performed using R version 4.3.0 with compounds satisfying VIP ≥ 1, |log2FC| > 0.5, and OAV ≥ 1. To assess the significance of differences among sample groups, one-way analysis of variance (ANOVA) was performed using R version 4.3.0, followed by Duncan’s multiple range test for post hoc comparisons, with a significance level set at *p* < 0.05. The assumptions of ANOVA were checked before statistical testing. Normality was assessed using Shapiro–Wilk test and homogeneity of variances was assessed using Levene’s test. The experiment design flowchart is shown in Figure 1.

## 3. Results

### 3.1. Sensory Quality of Jasmine Teas and Light Milk Teas

**Figure 2:**
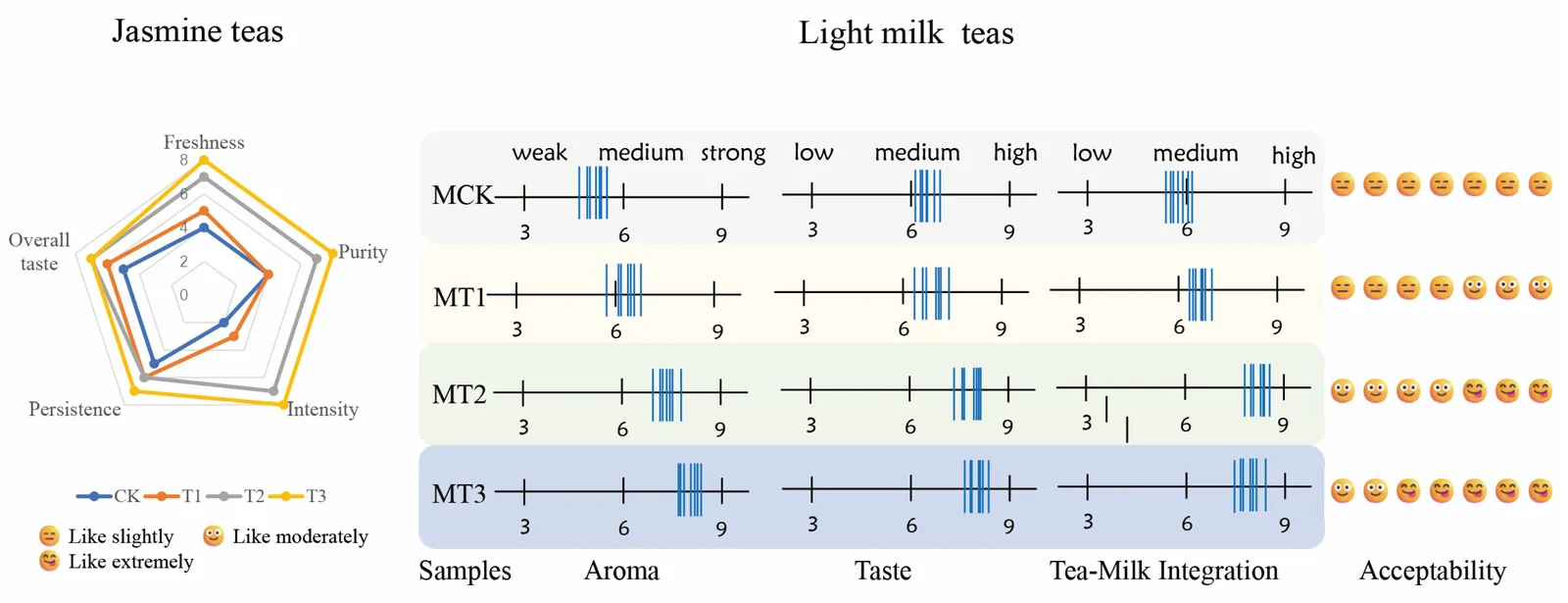
Sensory profiles of jasmine tea bases and corresponding light milk teas. Spider plot of sensory attributes (freshness, purity, intensity, persistence, and overall taste) for jasmine teas with increasing scenting cycles (CK, T1, T2, T3). Unstructured line scale scores of sensory attributes (aroma intensity, persistence, harmony; taste intensity, astringency, mellowness; tea–milk integration) for light milk teas (MCK, MT1, MT2, MT3). Panel-rated overall acceptability was defined as “Acceptability”, including like lightly, like moderately and like extremely. Different letters indicate significant differences (*p* < 0.05). (CK, control tea base; T1–T3, jasmine tea scented 1–3 times; MCK–MT3, corresponding milk tea products.).

The sensory profiles of jasmine tea bases and their corresponding light milk teas are shown in Figure 2. For jasmine tea, all evaluated attributes, including freshness, purity, intensity, persistence and overall taste, increased progressively from the control (CK) through the first (T1), second (T2), and third (T3) scenting treatments. T3 had the highest scores for all evaluated attributes among all treatments. This indicates a positive relationship between scenting frequency and perceived quality of jasmine teas. In the corresponding light milk teas, however, sensory differences were markedly reduced. While aroma attributes (intensity, persistence, harmony) and taste attributes (intensity, astringency, mellowness) still showed an upward trend from MCK to MT3, no significant differences were observed between MT2 and MT3. Notably, “tea-milk integration” received the highest ratings for both MT2 and MT3, and both achieved an overall acceptability level of “like extremely”. These improvements in key sensory dimensions were accompanied by higher overall acceptability, which shifted from “like slightly” for MCK to “like extremely” for MT2 and MT3. These results clearly demonstrate that although the scenting degree of the tea base significantly influences the final sensory acceptance of milk tea, further scenting did not produce a perceptible sensory advantage in the final milk tea product based on the sensory evaluation.

### 3.2. Volatile Profiles of Jasmine Teas and Light Milk Teas

#### 3.2.1. Volatile Compounds Identified in Jasmine Teas and Light Milk Teas

**Figure 3:**
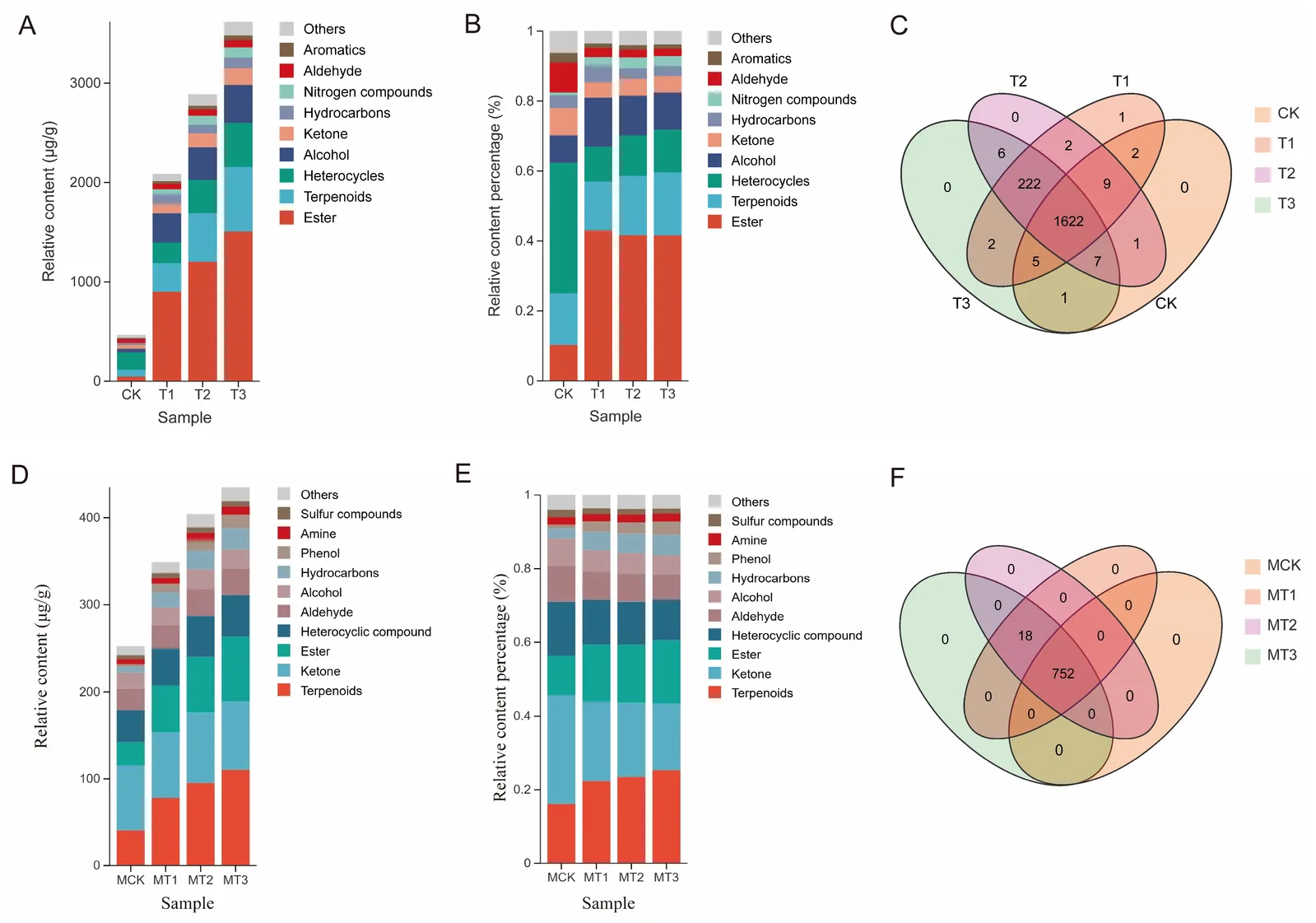
Variations in the volatile components of jasmine teas and light milk teas. Relative contents of VOCs in jasmine teas (**A**) and light milk teas (**D**); Relative content percentages of VOCs in jasmine teas (**B**) and light milk teas (**E**); Venn diagram analysis of VOCs in jasmine teas (**C**) and light milk teas (**F**) (CK, control tea base; T1–T3, jasmine tea scented 1–3 times; MCK–MT3, corresponding milk tea products).

A broad range of volatile compounds was observed in both jasmine tea bases and light milk teas, and these signals were further categorized into major chemical classes based on standard substances, RI comparison, and literature references (Figure 3). In jasmine teas, esters (18.04%), terpenoids (19.21%), heterocycles (11.07%), and alcohols (7.82%) were the dominant volatile classes (Table S1). Compared to the unscented base tea (CK), repeated scenting significantly increased the total volatile content and markedly elevated the proportion of esters, while reducing the relative abundance of heterocyclic compounds. For light milk teas, the detected VOCs comprised 5.20% acids, 9.75% alcohols, 7.54% aldehydes, 16.12% esters, 12.48% heterocycles, 13.78% terpenoids, 6.76% hydrocarbons and 11.83% ketones. Esters (16.12%), terpenoids (13.78%), ketones (11.83%), and heterocycles (12.48%) were the predominant volatile classes (Table S2). As the scenting cycles of the tea base increased, the proportions of esters and terpenoids in the corresponding milk tea showed a consistent upward trend, whereas the ketone proportion gradually declined.

The Venn diagram illustrates the distribution of shared and unique VOCs across samples (Figure 3C,F). In the tea bases, T2 and T3 shared the highest number of compounds (1857), indicating high chemical similarity. In the light milk teas, 752 VOCs were common to all samples. Only 18 compounds were uniquely detected in milk teas prepared from scented tea bases (MT1, MT2, MT3) and were absent in the milk tea prepared from the unscented base (MCK). Analysis revealed that these 18 unique compounds primarily belonged to the terpenoid, ester, and alcohol classes (Table S3). This convergence indicates that the milk system not only reduces the overall complexity of the volatile profile but also attenuates compositional differences originating from variations in tea base scenting intensity.

#### 3.2.2. Multivariate Analysis of Volatile Profiles in Jasmine Teas and Light Milk Teas

**Figure 4:**
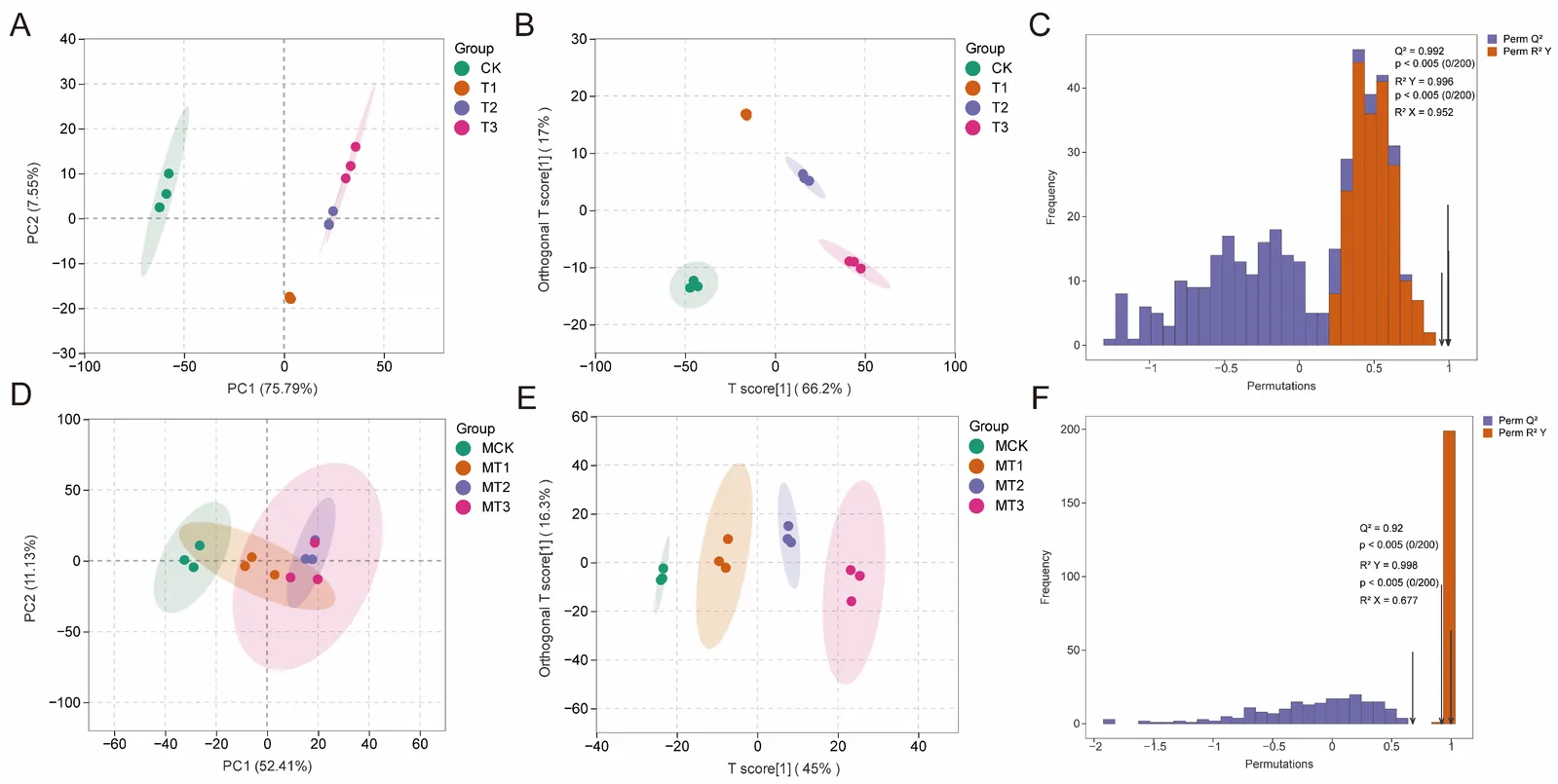
Principal component analysis of VOCs in jasmine teas (**A**) and light milk teas (**D**). Orthogonal partial least squares-discriminant analysis and R^2^Y and Q^2^ values of volatiles in jasmine teas (**B**,**C**) and light milk teas (**E**,**F**) (CK, control tea base; T1–T3, jasmine tea scented 1–3 times; MCK–MT3, corresponding milk tea products.).

Principal component analysis (PCA) was used to visualize overall metabolite differences among samples (Figure 4A,D). CK was clearly separated from the scented samples (T1–T3) along PC1 (75.79% variance). T1, T2, and T3 were further separated along PC2, although T2 and T3 were positioned close together, indicating highly similar volatile profiles. Similarly, in the corresponding milk tea samples (Figure 4D), PCA showed a similar but less pronounced separation pattern. MCK was distinctly separated from MT2 and MT3 along PC1 (52.41%). However, MT1, MT2 and MT3 were clustered together. This result indicates that the pronounced chemical differences inherited from their respective tea bases were substantially attenuated upon incorporation into the milk matrix. The attenuation likely results from matrix effects such as selective retention, phase partitioning, binding to milk proteins and lipids, and dilution.

To specifically resolve variance associated with the scenting cycles, supervised orthogonal partial least squares-discriminant analysis (OPLS-DA) was employed. In contrast to the unsupervised PCA, the OPLS-DA score plot (Figure 4B,E) demonstrated clear and systematic separation along the predictive component (t [1]), regardless of jasmine teas and light milk teas. All OPLS-DA models exhibited strong validity and predictive power (R^2^Y and Q^2^ values > 0.92) and passed permutation testing (*n* = 200, *p* < 0.01), confirming robustness and the absence of overfitting. The stark contrast between the unsupervised (PCA) and supervised (OPLS-DA) models provided direct chemical evidence that the volatile profile differences arising from tea base scenting are significant but are attenuated by the dominant and complex background of the milk and sugar system. This underscores the necessity of using supervised modeling to capture subtle target signals when analyzing flavor transfer in composite food systems.

#### 3.2.3. Analysis of Differential VOCs Among Jasmine Teas and Light Milk Teas

Differential compounds were screened from jasmine teas (T3 vs. T1, T2 vs. T1) and light milk teas (MT3 vs. MT1, MT2 vs. MT1) comparisons, respectively, using the criteria VIP ≥ 1, |log2FC| > 0.5 and *p* ≤ 0.05. The top 20 VOCs in each comparison are shown in Table 1. In the jasmine tea system, the relative contents of the key differential compounds consistently increased significantly with the number of scenting cycles. For example, the contents of methyl benzoate and benzyl acetate increased from 0.22 and 0.39 μg/g in CK to 231.12 and 304.11 μg/g in T3, respectively, and methyl anthranilate showed a similar marked increase from 0.35 μg/g in CK, demonstrating the highly efficient adsorption and enrichment of ester compounds during scenting. Similarly, linalool content increased dramatically from 1.02 μg/g in CK to 95.89 μg/g in T3 (a 94-fold increase). Indole and benzyl alcohol also exhibited substantial accumulation, rising from 1.29 μg/g to 227.26 μg/g and from 0.45 μg/g to 149.95 μg/g, respectively.

In the light milk tea system, the measured relative VOC contents were consistently lower than those in the corresponding tea bases due to methodological differences and the significant dilution/modifying effect of the milk matrix. Consequently, the increasing trends of most compounds were less pronounced in the milk system. However, key differential compounds, such as linalool, 6-camphenone, methyl anthranilate, and benzyl alcohol, still showed a consistent relative increase aligned with the scenting gradient of the source tea base (MT3 > MT2 > MT1 > MCK). This indicates that the “flavor imprint” of the jasmine teas was partially preserved and could systematically influence the chemical profile of their corresponding light milk teas.

### 3.3. Analysis of Aroma Contributors Among Jasmine Teas and Light Milk Teas

**Figure 5:**
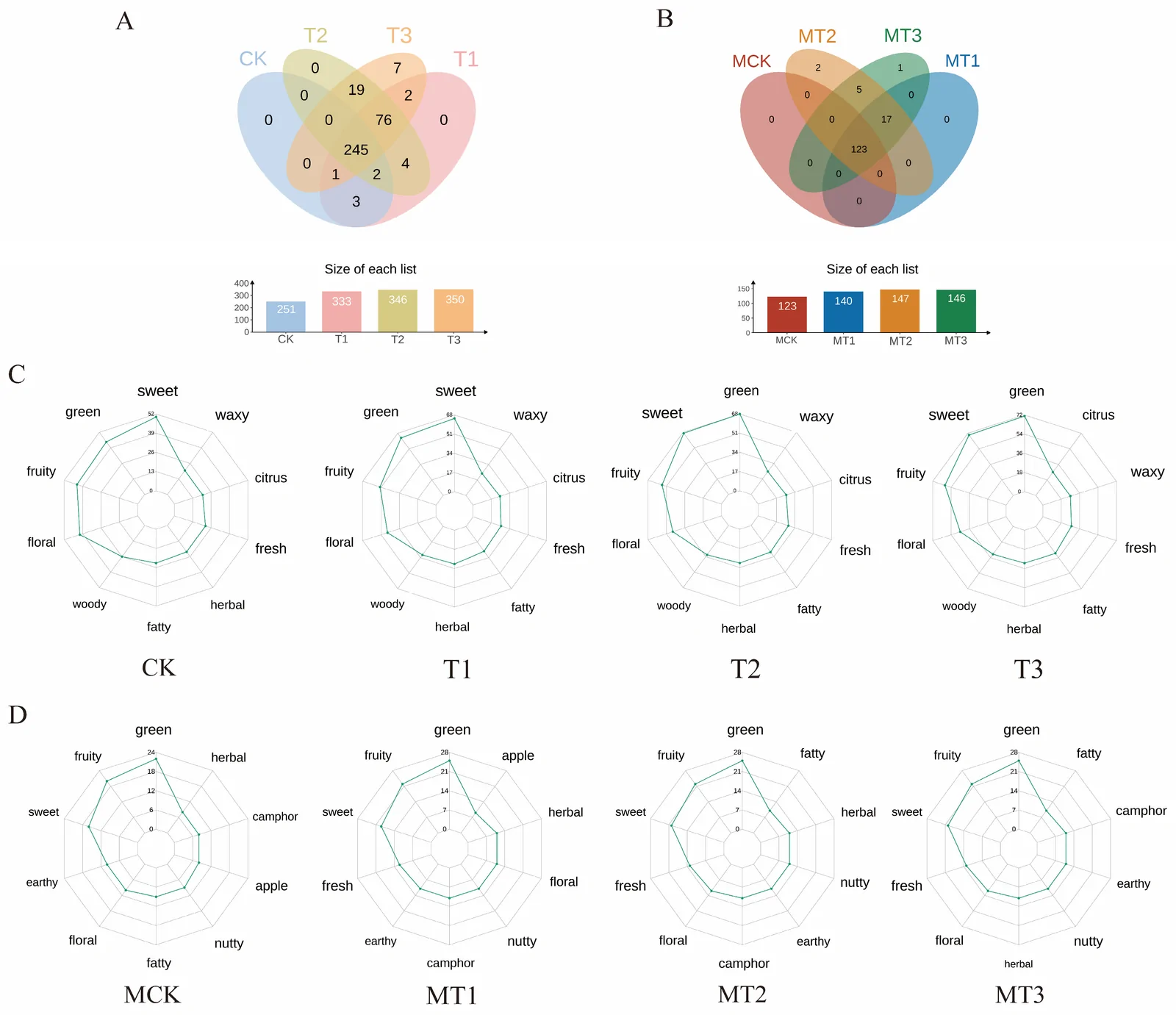
Key aroma-related metrics of jasmine teas and their corresponding milk teas. Venn Diagram of VOCs (ROAV > 1) in jasmine teas (**A**) and their corresponding milk teas (**B**), the flavor radar plots of jasmine teas (**C**) and their corresponding milk teas (**D**) (CK, control tea base; T1–T3, jasmine tea scented 1–3 times; MCK–MT3, corresponding milk tea products.).

To identify compounds making key contributions to the overall aroma, VOCs with an ROAV > 1 in at least one sample were analyzed. In jasmine teas, the numbers of such compounds were 251, 333, 346, and 350 for CK, T1, T2, and T3, respectively. The Venn diagram illustrated the distribution of VOCs with ROAV > 1 across jasmine tea samples (Figure 5A,B). No unique VOCs were detected in CK, T1 and T2. T3 contained 7 unique compounds. A total of 245 VOCs were shared across all four jasmine tea samples, and T2–T3 exhibited the most prominent co-occurrence (340 shared compounds), which is consistent with their clustered PCA distribution. In light milk teas, a total of 123 VOCs were shared across all milk tea samples, and MT2–MT3 shared the highest number of compounds (145), aligning with their overlapping PCA clustering. The associated bar plot indicates that the total count of VOCs in milk teas peaked at MT2 (127), followed by a slight decrease in MT3 (123).

The flavor radar plots show the dominant VOC-associated odor notes (Figure 5C,D). In the tea bases, sweet, green, fruity, and floral attributes gradually increased with more scenting cycles. In the light milk teas, the flavor profiles were dominated by green, fruity, and sweet notes, while the fresh floral attribute was significantly enhanced in MT1 through MT3 compared to MCK. Representative VOCs associated with these odor notes included pyrazine, 2-methoxy-3-(1-methylethyl)-, germacrene D, indole, benzyl alcohol, and (Z)-3-hexen-1-ol benzoate.

### 3.4. Identification of Key Aroma Contributors in Jasmine Tea and Light Milk Tea Systems

**Figure 6:**
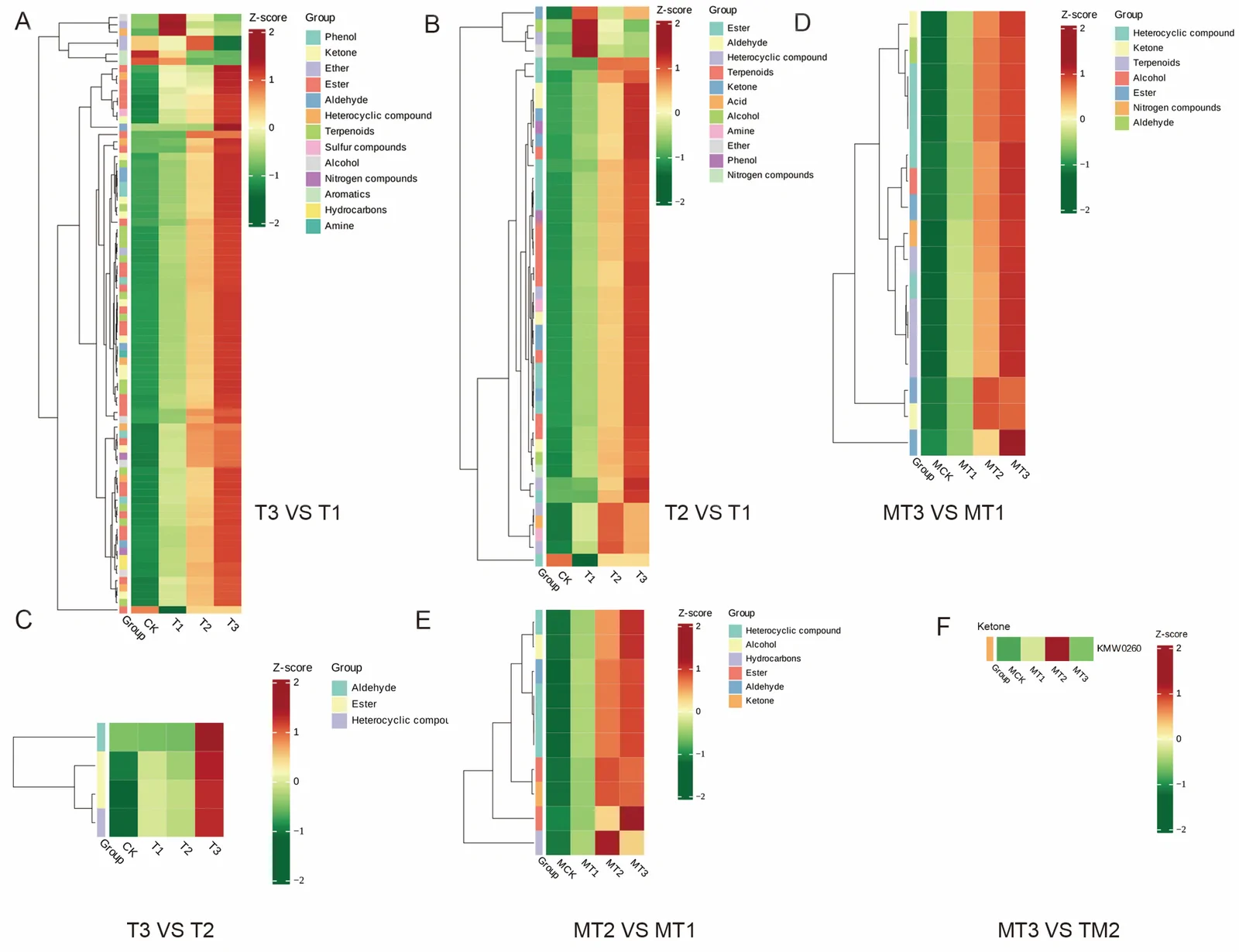
Cluster heatmap of volatiles screened with VIP ≥ 1, fold change > 2 or <0.5 and OAV ≥ 1 in jasmine teas (**A**–**C**) and in light milk teas (**D**–**F**). (CK, control tea base; T1–T3, jasmine tea scented 1–3 times; MCK–MT3, corresponding milk tea products.).

**Figure 7:**
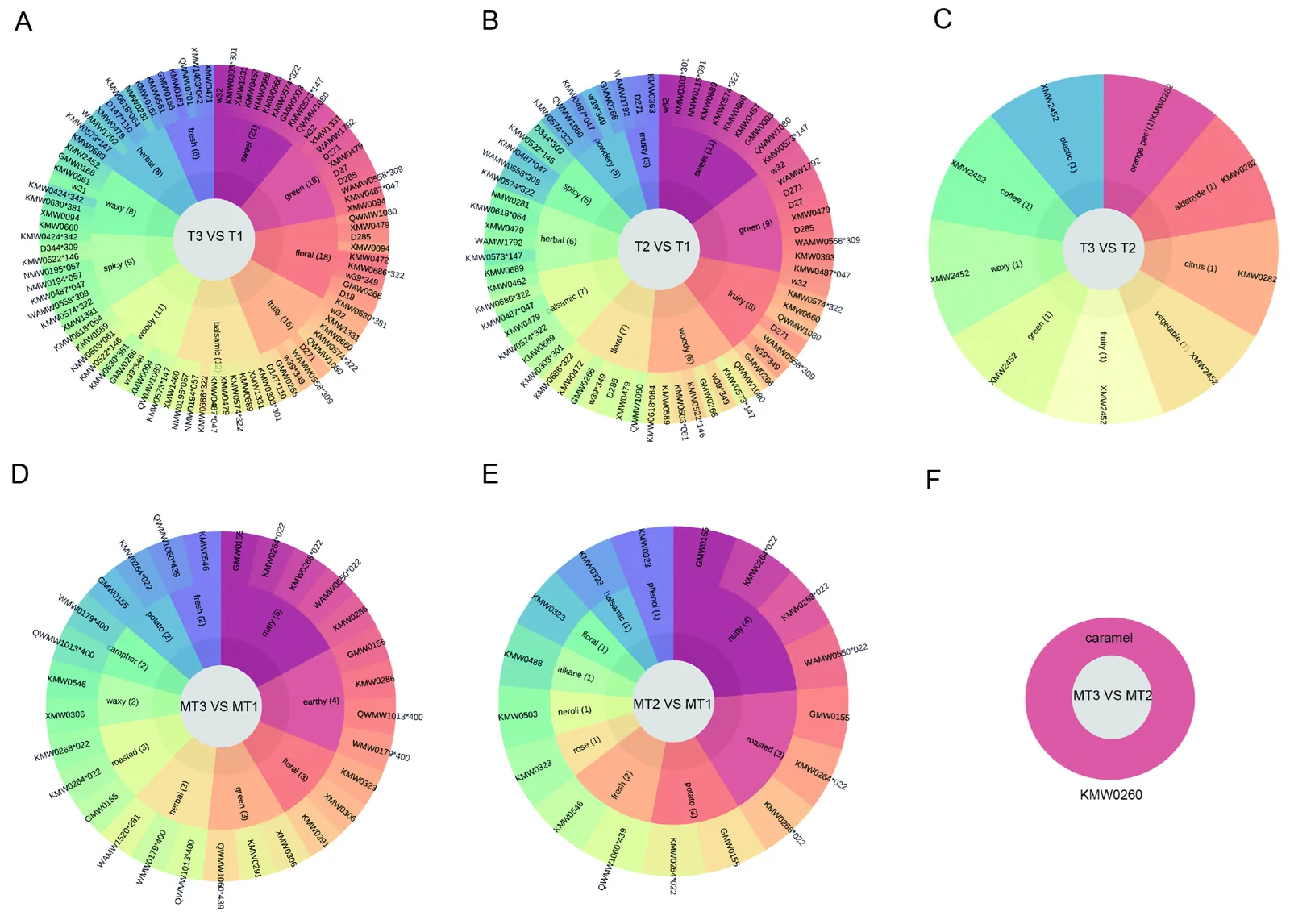
VOC-associated odor-note profiles of differential VOCs between different scenting jasmine teas and the light milk teas: (**A**). T3 vs. T1; (**B**). T2 vs. T1; (**C**). T3 vs. T2; (**D**). MT3 vs. MT1; (**E**). MT2 vs. MT1; (**F**). MT3 vs. MT2. (CK, control tea base; T1–T3, jasmine tea scented 1–3 times; MCK–MT3, corresponding milk tea products.).

To assess the contribution of compounds to the overall aroma, we screened compounds with VIP ≥ 1, fold change > 2 or <0.5 and OAV ≥ 1 and as criteria (Figure 6 and Figure 7, Table S4). In the jasmine tea system, the relative contents of the key differential aroma contributors exhibited a consistent upward trend with increasing scenting cycles. The most frequently annotated flavor attributes for these key compounds were sweet, green, floral, fruity and woody. Representative compounds contributing to these notes included pyrazine, 2-methoxy-3-(1-methylethyl)-, germacrene D, indole, benzyl alcohol, and (Z)-3-hexen-1-ol benzoate. In the comparison between T3 and T2, only four differential aroma compounds were identified, including nonanal, 1h-pyrrole-1-(2-furanylmethyl), 2,2,4-trimethyl-1,3-pentanediol diisobutyrate, and 1-octen-3-yl-n-propionate. These substances primarily impart orange peel, citrus, vegetable, and fruity aroma notes. A similar trend was observed in light milk teas, with the majority of key differential aroma contributors showing increasing relative content from MCK to MT3. However, this increasing trend was not pronounced in the comparison between MT3 and MT2. The most annotated flavor characteristics of these VOCs were nutty, earthy, floral, roasted. Representative compounds included (+)-dihydrocarvone, alpha-methyl-benzeneacetaldehyde, benzyl alcohol, linalool and methyl anthranilate. Notably, some aroma compounds exhibited a decreasing trend from MT2 to MT3, such as acetic acid, decyl ester and 3-Dodecanone, which are primarily associated with waxy, clean and fresh aroma notes. In the MT3 vs. MT2 comparison, only one compound, 2-Cyclopenten-1-one, 2-hydroxy-3,4-dimethyl- (KMW0260), was identified as a key differential contributor, showing an initial increase followed by a decrease, and is associated with a caramel-like aroma. An important observation is that certain compounds that increased substantially in the tea base due to scenting were not among the high-VIP compounds in the milk tea discrimination model. This suggests that these compounds may have been suppressed, bound, or transformed by milk and sugar components. Conversely, some compounds showing minor changes in the tea base became significant discriminators in milk tea, possibly due to synergistic or suppressive interactions with dairy constituents.

### 3.5. Comparative Analysis of Relative Responses of Common Differential VOCs Between Jasmine Tea and Light Milk Tea

To bridge the two independent datasets and reduce bias caused by methodological differences, the analysis focused on compounds that were consistently detected in both systems and showed significant variation along the scenting gradient (VIP > 1.0, *p* < 0.05, |log_2_FC| > 0.5) (Table 2). For these common differential compounds, “To examine how scenting-related changes observed in the tea base were reflected after milk-tea formulation, TF was calculated as the ratio of the fold change in the milk tea system to that in the tea base system: TF = (Fold change in light milk teas)/(Fold change in jasmine teas). Fold change was calculated relative to the first scenting level (MT3/MT1 vs. T3/T1 and MT2/MT1 vs. T2/T1). Accordingly, a higher TF indicates that the relative change associated with scenting was more strongly maintained or amplified in the milk-tea system compared with the tea-base system. The compounds exhibiting the highest TF values in the MT2/MT1 and MT3/MT1 comparisons are listed in Table 2. Notably, esters and ketones constituted a substantial proportion of the high-TF compounds. In the MT2/MT1 comparison, esters accounted for 30% (3 out of the top 10), and ketones were also prominent. Similarly, in the MT3/MT1 comparison, esters were again strongly represented. This suggests that esters and ketones were frequently represented among compounds with relatively high TF values, indicating that scenting-related changes in these compounds remained comparatively pronounced in the milk-tea system under the conditions investigated. The highest TF values were observed for Acetic acid, decyl ester (TF = 2.13) and 3-Dodecanone (TF = 2.08) in the MT2/MT1 comparison, and for Methyl anthranilate (TF = 2.39) in the MT3/MT1 comparison. These exceptionally high TF values indicate that these compounds are not merely transferred but are potentially enhanced in the milk tea system, possibly through precursor conversion or specific interactions with dairy components. Notably, a subset of compounds, including methyl anthranilate, linalool, butyl caprylate, acetic acid, decyl ester, 3-dodecanone, 1-(2-pyridinyl) ethenone, benzyl alcohol, and pyrazine, 2-methoxy-3-(1-methylethyl)-, appeared among the high-TF compounds in both comparisons. Thereinto, methyl anthranilate demonstrated higher transferred characteristics with the increased scenting cycles tea base. Their consistent appearance identifies them as “core transfer compounds” that stably exhibit high transfer efficiency regardless of the scenting intensity of the source tea base. These compounds are likely contributors to the characteristic flavor profile of jasmine milk tea.

### 3.6. Applicability Assessment of the Jasmine Tea Flavor (JTF) Index in Light Milk Tea Systems

The JTF index was deemed as the index for the quality evaluation of jasmine tea, which is calculated by the peak area percentage of α-farnesene, (Z)-3-hexenyl benzoate, methyl anthranilate, and indole relative to that of linalool. In this study, the JTF index exhibited a continuous and significant upward trend from CK to T3, with T3 showing the highest value (Table 3). This result supports the progressive improvement of aroma quality with increasing scenting cycles. However, the applicability of the JTF index was limited in the complex matrix of light milk tea (Table S3). Among the five compounds constituting the JTF index, only linalool and methyl anthranilate were consistently detected and quantifiable in light milk tea samples. The key components α-farnesene, (Z)-3-hexenyl benzoate, and indole were undetectable, rendering the conventional JTF index inapplicable for the aroma quality evaluation of light milk teas. This limitation underscores the necessity of developing a new, matrix-specific evaluation framework for tea-based beverage systems. Based on the identification of “core transfer compounds,” we propose that compounds with high and stable transfer efficiency, such as methyl anthranilate, linalool and benzyl alcohol, could serve as potential key chemical indicators for assessing the quality of light milk tea products.

## 4. Discussion

### 4.1. Sensory Convergence in Milk Tea Despite Divergent Tea Base Profiles

Previous studies on jasmine tea have demonstrated that aroma composition can continue to evolve with increasing scenting cycles, with additional scenting rounds contributing to further accumulation or transformation of characteristic volatile compounds, including more than three cycles [11,17]. In the present study, T2 and T3 also differed chemically and sensorially as pure teas. However, one of the most interesting findings is the sensory convergence between light milk teas prepared from two- (MT2) and three-scented (MT3) tea bases. This convergence occurred despite their pure tea counterparts (T2 vs. T3) showing distinct chemical and sensory differences. Several factors may contribute to this phenomenon. First, this attenuation can be mechanistically explained by selective interactions between tea volatiles and dairy components, which govern aroma release, stability, and perception in complex food systems [31]. Second, the conversion of jasmine tea into light milk tea involves substantial dilution of tea-derived volatiles within the final beverage formulation, potentially reducing the perceptual contribution of incremental aroma increases from higher scenting cycles. Therefore, the limited sensory benefit of the third cycle observed in this study should be understood within the specific formulation, flower-to-tea ratio, drying temperature, storage duration., milk composition, and panel conditions tested, rather than as a general conclusion that three scenting cycles are unnecessary for all jasmine milk tea products. Our VOC data support that esters and ketones, which have higher hydrophobicity, exhibited higher transfer factors (TF > 1), suggesting preferential retention in the lipid phase. Conversely, hydrophilic or protein-binding compounds such as indole and certain heterocyclics were undetectable in milk tea, likely due to irreversible binding or degradation. This selective retention reshapes the aroma profile. While the pure tea displays a complex, nuanced aroma, the milk tea system emphasizes a streamlined, “sweet-creamy-floral” profile driven by stable esters and terpenoids.

### 4.2. The Application of the JTF Index in Jasmine Teas and Light Milk Teas

Our study empirically validates the limitation of existing tea-centric quality indices in complex food systems. The established JTF index is defined as the ratio of peak area percentage of α-farnesene, (Z)-3-hexenyl benzoate, methyl anthranilate, and indole to that of linalool [16]. While this index has demonstrated effectiveness for pure tea, it failed to be directly applicable to light milk teas because key index compounds like α-farnesene and indole were undetectable or were substantially suppressed in the dairy matrix.

This phenomenon reflects a fundamental matrix-driven limitation rather than a deficiency of analytical detection alone, indicating that aroma evaluation systems developed for single-phase tea infusions may not be transferable to multiphase emulsion beverages. Consequently, there is a need to redefine quality evaluation frameworks for tea-based dairy systems based on matrix-compatible aroma behavior rather than absolute compositional similarity. In this context, we identified a subset of compounds—methyl anthranilate, linalool, benzyl alcohol, butyl caprylate, and several ketones (e.g., 3-dodecanone)—that exhibited high Transfer Factors (TF > 1). Notably, these compounds not only demonstrated efficient transfer from the tea matrix into the dairy system but also retained strong discriminative potential among different scenting-cycle treatments. Methyl anthranilate and linalool, well-established key floral aroma contributors in jasmine tea, possess moderate hydrophobicity that facilitates partitioning into the protective milk fat phase rather than engaging in strong binding with casein proteins [17]. Butyl caprylate, with its highly lipophilic nature, is likely fully incorporated into lipid droplets, acting as a reservoir for sustained release [32]. Meanwhile, the amphiphilic character of benzyl alcohol may position it at the oil–water interface, enabling rapid release upon consumption [33]. In addition, the disappearance of classical JTF marker compounds (e.g., α-farnesene and indole) can be further explained by their high susceptibility to matrix-induced transformation processes, including oxidative degradation in lipid environments, protein binding within casein micelles, and reduced volatility due to emulsified system constraints. These combined physicochemical effects lead to their loss of detectability and render them unsuitable as stable indicators in dairy-based systems.

Collectively, these findings demonstrate that the original JTF index is not inherently invalid, but rather system-specific, being optimized for aqueous tea infusions rather than emulsified beverage matrices. We therefore propose that these “core transfer compounds” with high TF values and stable detectability across matrices represent more robust chemical markers for evaluating jasmine tea quality in milk tea applications, shifting the focus from total aroma abundance to matrix-relevant aroma performance.

### 4.3. Mechanistic Insights into Flavor Transfer: Esters and Ketones as Stable Carriers

The analysis of TF provided a quantitative insight into flavor fate during beverage production. The prominence of esters and ketones among high-TF compounds is mechanistically significant. Esters, being relatively hydrophobic, may preferentially partition into the milk fat phase, which can act as a reservoir, slowing their release and potentially protecting them from degradation [34]. This is consistent with our observation that esters showed a high retention. Conversely, hydrophilic or protein-binding compounds such as indole may be adsorbed by casein or undergo degradation, explaining their undetectability in milk tea. This selective process reshapes the final aroma profile: the fresh, green top notes prominent in pure tea may be subdued, while the sweet, floral, and fruity notes contributed by stable esters and some terpenoids (like linalool) become more defining in the milk tea. The differential contribution of jasmine floral volatiles to tea aroma has been well documented [13], with ester compounds such as benzyl acetate and methyl salicylate identified as key contributors. Our study extends this understanding by demonstrating that these same esters exhibit excellent stability in dairy matrices, making them important carriers of milk tea aroma. Furthermore, recent metabolomics studies have revealed dynamic changes in volatile metabolites during the scenting process [35], and our work adds another layer by revealing the secondary dynamic changes that occur during the tea-to-milk tea transformation. Importantly, these mechanisms are proposed as plausible explanations rather than demonstrated conclusions. Direct evidence for protein binding, lipid partitioning, or interfacial retention was not obtained in this study. Future work using model dairy systems and binding assays is needed to confirm these mechanisms.

### 4.4. Industrial Implications and Future Research Directions

From an industrial perspective, our findings challenge the conventional assumption that a higher-grade tea base invariably yields a superior milk tea. While traditional jasmine tea production typically involves multiple scenting cycles to maximize aroma intensity [36], our results suggest that the marginal or insignificant sensory gain from a third scenting cycle was observed within the milk matrix. In addition, this study establishes a flavor-transfer framework that integrates traditional tea processing parameters with modern beverage matrix effects. Unlike previous studies that focused solely on tea volatiles or beverage sensory properties in isolation, our approach systematically links gradient scenting, volatile accumulation, matrix-driven selectivity, and final sensory perception. This framework advances the field in two key ways: (1) it shifts the quality paradigm from total aroma abundance in tea bases to matrix-relevant aroma performance, identifying “core transfer compounds” as stable markers for beverage quality; and (2) it provides a methodology (TF analysis combined with OPLS-DA and ROAV) for predicting flavor behavior in composite systems. However, this study has several limitations and future directions. We used a single standardized milk tea formula; different fat contents, protein sources (dairy vs. plant-based), or sweetener levels could interact differently with tea volatiles. Second, our analysis focused on headspace volatiles; exploring the role of non-volatile tea components in modulating aroma binding and astringency balance would provide a more comprehensive understanding. Addressing these aspects will further solidify the scientific foundation for the rational design of premium, consistent, and appealing tea-based beverages.

## 5. Conclusions

This study demonstrates that repeated scenting progressively enhanced the aroma intensity and volatile complexity of jasmine tea bases. However, the additional aroma differences between two- and three-scented teas were substantially attenuated after incorporation into the standardized milk tea matrix under the conditions investigated here. The results indicate that aroma enhancement achieved during tea processing is not necessarily expressed proportionally in the final dairy beverage, highlighting the importance of considering matrix effects when evaluating tea aroma quality for beverage applications. The milk tea matrix was associated with compound-dependent changes in volatile detectability and relative responses, resulting in a more convergent aroma profile compared with the corresponding tea bases. The conventional JTF index originally developed for jasmine tea evaluation showed limited applicability in the milk tea system due to the reduced detectability of several indicator compounds. Instead, matrix-stable core transfer compounds such as methyl anthranilate, linalool, and benzyl alcohol remained consistently detectable across the two matrices and may therefore serve as candidate matrix-robust markers for jasmine milk tea evaluation. The practical expression of tea-derived aroma in milk-based beverages is likely influenced by multiple factors, including tea base characteristics, jasmine flower quality, dairy composition, sweetener level, formulation design, and cost considerations. Although interactions between volatile compounds and milk components may contribute to the observed differences, these mechanisms were inferred from volatile profiles and previous studies and require further validation through direct measurements of compound partitioning, protein binding, and aroma release behavior. Overall, this study provides insights into how beverage matrices modulate the expression of tea-derived aroma characteristics and emphasizes that tea raw materials should be evaluated within the context of their intended product formulation rather than solely based on their aroma performance in the original tea system.

## Figures and Tables

**Table 1:** Top 20 VOCs screened from the tea base (T3 vs. T1, T2 vs. T1) and light milk tea (MT3 vs. MT1, MT2 vs. MT1) comparisons, respectively (VIP ≥ 1, |log2FC| > 0.5, and *p* < 0.05). The asterisk means the same compounds sharded by tea base (T3 vs. T1, T2 vs. T1) vs light milk tea (MT3 vs. MT1, MT2 vs. MT1). (Different lower-case letters indicate significant difference, *p* < 0.05).

NO.	Compounds	Class I	CAS	CK (μg/g)	T1 (μg/g)	T2 (μg/g)	T3 (μg/g)	Odor
1	Linalool *	Terpenoids	78-70-6	1.02 ± 0.08 d	55.81 ± 1.53 c	79.51 ± 3.32 b	95.89 ± 2.65 a	floral, green
2	Germacrene D	Terpenoids	23986-74-5	0.25 ± 0.02 d	4.68 ± 0.14 c	10.87 ± 0.36 b	16.39 ± 1.52 a	woody, spice
3	alpha.-Farnesene	Terpenoids	502-61-4	0.06 ± 0.01 d	11.74 ± 0.18 c	15.49 ± 0.21 b	19.99 ± 1.07 a	citrus, herbal, lavender, bergamot, myrrh, neroli, green
4	Naphthalene, 1,2,3,5,6,8a-hexahydro-4,7-dimethyl-1-(1-methylethyl)-, (1S-cis)-	Terpenoids	483-76-1	0.9 ± 0.06 d	4.56 ± 0.17 c	9.27 ± 0.09 b	13.11 ± 1.40 a	thyme, herbal, woody, dry
5	1H-Benzocycloheptene, 2,4a,5,6,7,8-hexahydro-3,5,5,9-tetramethyl-, (R)-	Terpenoids	1461-03-6	0.04 ± 0.01 d	5.10 ± 0.21 c	8.29 ± 0.28 b	11.22 ± 0.92 a	\
6	2,3-Dimethyl-5-n-propylpyrazine	Heterocyclics	32262-98-9	<0.01 d	2.50 ± 0.07 c	5.13 ± 0.19 a	4.19 ± 0.11 b	\
7	Pyrazine, 2-methoxy-3-(1-methylethyl)-	Heterocyclics	25773-40-4	1.02 ± 0.10 c	5.57 ± 0.13 b	10.64 ± 0.35 a	11.09 ± 0.22 a	beany, pea, earthy, chocolate, nutty
8	Indole	Heterocyclics	120-72-9	1.29 ± 0.17 d	9.62 ± 0.08 c	132.41 ± 3.65 b	227.26 ± 6.6 a	animalic, floral, moth, mothball, fecal, naphthalene
9	Oxirane, tetradecyl-	Heterocyclics	7320-37-8	0.37 ± 0.02 d	4.49 ± 0.14 c	10.34 ± 0.51 b	15.36 ± 0.92 a	\
10	(3R,6S)-2,2,6-Trimethyl-6-vinyltetrahydro-2H-pyran-3-ol	Heterocyclics	39028-58-5	3.86 ± 0.46 c	4.37 ± 0.20 b	4.76 ± 0.18 a	4.86 ± 0.03 a	woody
11	3-Hexen-1-ol, acetate, (Z)-	Ester	3681-71-8	0.05 ± 0.00 c	50.97 ± 1.47 a	39.12 ± 1.54 b	51.5 ± 0.92 a	fresh, green, sweet, fruity, banana, apple, grassy
12	Benzoic acid, methyl ester	Ester	93-58-3	0.22 ± 0.02 d	124.27 ± 3.07 c	219.93 ± 8.16 b	231.12 ± 4.66 a	phenol, wintergreen, almond, floral, canga
13	Acetic acid, phenylmethyl ester *	Ester	140-11-4	0.39 ± 0.13 d	219.23 ± 4.68 c	238.17 ± 8.86 b	304.11 ± 7.51 a	sweet, floral, fruity, jasmin, fresh
14	Methyl anthranilate *	Ester	134-20-3	0.35 ± 0.26 d	85.43 ± 1.3 c	121.96 ± 4.79 b	151.22 ± 2.5 a	fruity, grape, orange, flowery, neroli
15	Methyl salicylate	Ester	119-36-8	0.41 ± 0.04 d	14.52 ± 0.22 c	19.94 ± 0.74 b	21.98 ± 0.42 a	caramel, pepperminty
16	Benzenemethanol, 4-methyl-α-[(1R)-1-methyl-2-propen-1-yl]-, (αR)-rel-	Alcohol	83173-76-6	0.07 ± 0.00 d	0.82 ± 0.02 c	2.23 ± 0.06 b	3.40 ± 0.29 a	\
17	Benzyl alcohol *	Alcohol	100-51-6	0.45 ± 0.04 d	72.29 ± 0.71 c	131.86 ± 9.97 b	149.95 ± 3.91 a	floral, rose, phenol, balsamic
18	11,11-Dimethyl-4,8-dimethylenebicyclo[7.2.0]undecan-3-ol	Alcohol	79580-01-1	0.28 ± 0.03 d	1.00 ± 0.03 c	1.82 ± 0.08 b	2.54 ± 0.16 a	\
19	2-Propen-1-ol, 3-phenyl-	Alcohol	104-54-1	0.07 ± 0.00 d	0.91 ± 0.35 c	1.80 ± 0.16 b	2.36 ± 0.05 a	sweet, balsamic, hyacinth, spicy, green, powdery, cinnamon
20	Benzenepropanol, 4-hydroxy-3-methoxy-	Alcohol	2305-13-7	0.17 ± 0.02 d	0.54 ± 0.01 c	1.01 ± 0.49 b	1.43 ± 0.10 a	\
NO.	Compounds	Class I	CAS	MCK (μg/mL)	MT1 (μg/mL)	MT2 (μg/mL)	MT3 (μg/mL)	Odor
1	6-Octen-1-ol, 7-methyl-3-methylene-	Terpenoids	13066-51-8	1.50 ± 0.07 b	1.53 ± 0.16 ab	1.67 ± 0.09 a	1.59 ± 0.08 ab	\
2	(+)-Dihydrocarvone	Terpenoids	5524-05-0	0.40 ± 0.01 d	2.05 ± 0.18 c	3.97 ± 0.20 b	4.81 ± 0.06 a	herbal, minty, menthol
3	Fenchone	Terpenoids	1195-79-5	0.15 ± 0.01 d	0.64 ± 0.04 c	1.06 ± 0.05 b	1.31 ± 0.06 a	herbal, cedar leaf, bitter, thuja, camphor, earthy, woody
4	Linalool *	Terpenoids	78-70-6	0.15 ± 0.00 d	0.64 ± 0.04 c	1.05 ± 0.04 b	1.31 ± 0.06 a	floral, green
5	7-Octen-4-ol, 2-methyl-6-methylene-, (S)-	Terpenoids	35628-05-8	0.21 ± 0.01 d	0.37 ± 0.02 c	0.52 ± 0.03 b	0.59 ± 0.03 a	\
6	2H-Pyran-2-one, tetrahydro-6-methyl-	Ketone	823-22-3	0.30 ± 0.01 c	0.36 ± 0.03 b	0.43 ± 0.02 a	0.44 ± 0.03 a	creamy, fruity, coconut
7	2-Pyrrolidinone, 1-ethenyl-	Ketone	88-12-0	0.06 ± 0.00 d	0.12 ± 0.01 c	0.17 ± 0.01 b	0.20 ± 0.12 a	\
8	2,6,6-Trimethylbicyclo[3.2.0]hept-2-en-7-one	Ketone	4613-37-0	0.13 ± 0.00 d	0.26 ± 0.02 c	0.39 ± 0.02 b	0.44 ± 0.02 a	\
9	Camphenone, 6-	Ketone	55659-42-2	0.26 ± 0.01 d	1.54 ± 0.09 c	2.62 ± 0.13 b	3.28 ± 0.15 a	\
10	Ethanone, 2-hydroxy-1-phenyl-	Ketone	582-24-1	0.02 ± 0.01 d	1.21 ± 0.08 c	1.47 ± 0.06 b	1.97 ± 0.06 a	\
11	2-Acetyl-5-methylfuran	Heterocyclics	1193-79-9	0.38 ± 0.03 c	0.51 ± 0.04 b	0.70 ± 0.05 a	0.74 ± 0.03 a	strong, musty, nutty, hay, coconut, coumarin, milky
12	Pyrazine, 2-methyl-6-propyl-	Heterocyclics	29444-46-0	0.17 ± 0.00 dc	0.21 ± 0.01 b	0.25 ± 0.01 a	0.26 ± 0.02 a	burnt, butterscotch
13	Pyridine, 5-ethyl-2-methyl-	Heterocyclics	104-90-5	0.05 ± 0.01 d	0.78 ± 0.03 c	1.87 ± 0.11 b	2.29 ± 0.05 a	nutty, strong, raw, potato, roasted, earthy
14	Pyrazine, ethenyl-	Heterocyclics	4177-16-6	0.03 ± 0.00 d	0.09 ± 0.00 c	0.15 ± 0.00 b	0.17 ± 0.01 a	green, burnt, nutty
15	Pyrazine, 2,3-diethyl-5-methyl-	Heterocyclics	18138-04-0	0.01 ± 0 d	4.19 ± 0.31 c	5.26 ± 0.27 b	7.27 ± 0.24 a	musty, nut skin, earthy, roasted, hazelnut, toasted, potato, dusty, foliage, vegetable
16	2-Butenoic acid, 2-methyl-, 2-methylpropyl ester, (E)-	Ester	61692-84-0	1.15 ± 0.04 c	1.41 ± 0.10 b	1.7 ± 0.09 a	1.76 ± 0.11 a	herbal, pungent
17	4-Heptanol, 2,6-dimethyl-, acetate	Ester	10250-45-0	1.14 ± 0.04 c	1.68 ± 0.11 b	2.19 ± 0.11 a	2.37 ± 0.13 a	herbal, rhubarb, floral, lilac, banana
18	Methyl anthranilate *	Ester	134-20-3	0.04 ± 0.00 d	0.22 ± 0.02 c	0.54 ± 0.05 b	0.91 ± 0.06 a	fruity, grape, orange, flowery, neroli
19	Acetic acid, 4-methylphenyl ester	Ester	140-39-6	0.04 ± 0.01 d	10.39 ± 0.74 c	12.86 ± 0.67 b	17.67 ± 0.52 a	narcissus, phenol, animalic
20	Acetic acid, phenylmethyl ester *	Ester	140-11-4	0.02 ± 0.01 d	4.75 ± 0.33 c	5.84 ± 0.29 b	7.89 ± 0.23 a	sweet, floral, fruity, jasmin, fresh
21	Triethylene glycol	Alcohol	112-27-6	1.11 ± 0.05 a	1.05 ± 0.09 a	1.15 ± 0.07 a	0.87 ± 0.06 b	\
22	3-Octanol, 3,7-dimethyl-	Alcohol	78-69-3	0.13 ± 0.00 bc	0.14 ± 0.01 b	0.16 ± 0.01 a	0.16 ± 0.01 ab	lilac, oily, lily, tea, floral, bois de rose, woody
23	Benzyl alcohol *	Alcohol	100-51-6	0.12 ± 0.02 d	1.17 ± 0.07 c	2.70 ± 0.15 b	3.28 ± 0.06 a	floral, rose, phenol, balsamic
24	2H-Pyran-2-methanol, tetrahydro-	Alcohol	100-72-1	0.04 ± 0.00 c	0.27 ± 0.01 a	0.21 ± 0.01 d	0.29 ± 0.01 a	\
25	1,5-Pentanediol	Alcohol	111-29-5	0 ± 0 c	0.28 ± 0.01 a	0.21 ± 0.01 b	0.30 ± 0.14 a	\

**Table 2:** The transfer factor (TF) of top 10 VOCs screened from MT2 vs. MT1 and MT3 vs. MT1, respectively (VIP ≥ 1, |log2FC| > 0.5, and *p* < 0.05). The asterisk means the same compounds shared by MT2 vs. MT1 and MT3 vs. MT1. A TF > 1.5 indicates enhanced transfer from jasmine tea to the light milk tea (Type E: Enhanced aroma); 0.7 < TF < 1.5 indicates stable transfer (Type S: Stable aroma).

No.	Index	Compounds	Class I	MT2/MT1			rOAV	T2/T1			rOAV	TF	Type	Odor
				VIP	FC	Tend	MT2	VIP	FC	Tend	T2			
1	KMW0546	Acetic acid, decyl ester *	Ester	1.10	2.12	up	1.50	0.11	0.99	insig	15.23	2.13	E	waxy, clean, fresh, laundered cloth, cloth, citrus, soapy
2	WAMW0663	3-Dodecanone *	Ketone	1.07	2.07	up	1.84	0.09	1.00	insig	17.49	2.08	E	-
3	XMW0089	4-Decenal, (E)-	Aldehyde	1.06	1.62	up	3.12	1.01	0.90	insig	7.58	1.81	E	fresh, aldehydic, citrus, orange, mandarin, tangerine, green, fatty
4	KMW0503	Methyl anthranilate *	Ester	1.37	2.45	up	180.87	1.18	1.45	up	40,654.48	1.68	E	fruity, grape, orange, flowery, neroli
5	KMW0488	Tridecane	Hydrocarbons	1.09	2.13	up	1.00	1.07	1.34	insig	2.86	1.59	E	alkane
6	KMW0494	Decanoic acid, methyl ester	Ester	1.12	1.97	up	7.39	1.14	1.39	insig	39.83	1.41	S	oily, wine, fruity, floral
7	KMW0203	Ethanone, 1-(2-pyridinyl)- *	Ketone	1.35	1.97	up	1.67	1.18	1.61	up	27.72	1.22	S	popcorn, heavy, corn, chip, fatty, tobacco
8	KMW0323	Benzyl alcohol *	Alcohol	1.37	2.24	up	27.03	1.18	1.86	up	1318.61	1.21	S	floral, rose, phenol, balsamic
9	KMW0291	Linalool *	Terpenoids	1.37	1.60	up	174.31	1.18	1.45	up	13,252.02	1.10	S	floral, green
10	KMW0286	Pyrazine, 2-methoxy-3-(1-methylethyl)- *	Heterocyclics	1.37	1.66	up	24,583.04	1.18	1.95	up	5,319,725.80	0.85	S	beany, pea, earthy, chocolate, nutty
**No.**	**Index**	**Compounds**	**Class I**	**MT3/MT1**			**rOAV**	**T3/T1**			**rOAV**	**TF**	**Type**	**Odor**
				**VIP**	**FC**	**Tend**	**MT3**	**VIP**	**FC**	**Tend**	**T3**			
1	KMW0503	Methyl anthranilate *	Ester	1.51	4.27	up	303.81	1.15	1.79	up	50,405.99	2.39	E	fruity, grape, orange, flowery, neroli
2	WAMW0663	3-Dodecanone *	Ketone	1.18	2.01	up	1.73	0.96	1.14	insig	20.25	1.76	E	-
3	KMW0546	Acetic acid, decyl ester *	Ester	1.21	2.02	up	1.38	0.98	1.15	insig	17.77	1.76	E	waxy, clean, fresh, laundered cloth, cloth, citrus, soapy
4	KMW0323	Benzyl alcohol *	Alcohol	1.51	2.83	up	32.85	1.15	2.10	up	1499.51	1.35	S	floral, rose, phenol, balsamic
5	KMW0203	Ethanone, 1-(2-pyridinyl)- *	Ketone	1.50	2.29	up	1.88	1.13	1.86	up	32.13	1.23	S	popcorn, heavy, corn, chip, fatty, tobacco
6	KMW0350	Acetic acid, phenylmethyl ester	Ester	1.51	1.68	up	21.69	1.15	1.40	insig	835.47	1.20	S	sweet, floral, fruity, jasmin, fresh
7	KMW0291	Linalool *	Terpenoids	1.51	2.07	up	217.50	1.15	1.74	up	15,981.90	1.19	S	floral, green
8	QWMW1099	5-Octen-1-ol, (Z)-	Alcohol	1.51	1.50	insig	21.73	1.14	1.34	insig	157.24	1.12	S	green, melon, watery, watermelon, earthy, mushroom, violet, leafy, fishy, soapy
9	KMW0286	Pyrazine, 2-methoxy-3-(1-methylethyl)- *	Heterocyclics	1.51	2.15	insig	30,653.17	1.15	2.01	up	5,545,618.55	1.07	S	beany, pea, earthy, chocolate, nutty
10	KMW0278	Ethanone, 1-(2-thienyl)-	Ketone	1.51	1.78	insig	195.70	1.15	1.83	up	7623.38	0.98	S	sulfury, nutty, hazelnut, walnut

**Table 3:** The value of JTF and the content of related compounds in jasmine teas and light milk teas. (Different lower-case letters indicate significant difference, *p* < 0.05).

No.	Compounds	Class I	CAS	Jasmine Tea Samples (μg/g)			
				CK	T1	T2	T3
1	Linalool	Terpenoids	78-70-6	1.02 ± 0.08 d	55.81 ± 1.53 c	79.51 ± 3.32 b	95.89 ± 2.65 a
2	Indole	Heterocyclics	120-72-9	1.29 ± 0.17 d	9.62 ± 0.08 c	132.41 ± 3.65 b	227.26 ± 6.60 a
3	Methyl anthranilate	Ester	134-20-3	0.35 ± 0.26 d	85.43 ± 1.30 c	121.96 ± 4.79 b	151.22 ± 2.50 a
4	.alpha.-Farnesene	Terpenoids	502-61-4	0.06 ± 0.01 d	11.74 ± 0.18 c	15.49 ± 0.21 b	19.99 ± 1.07 a
5	3-Hexen-1-ol, benzoate, (Z)-	Ester	25152-85-6	0.35 ± 0.03 d	185.04 ± 2.49 c	286.70 ± 8.95 b	378.99 ± 14.68 a
6	JTF	/	/	2.01 ± 0.23 d	5.23 ± 0.09 c	7.00 ± 0.11 b	8.11 ± 0.22 a
No.	Compounds	Class I	CAS	Light milk tea samples (μg/mL)			
				MCK	MT1	MT2	MT3
7	Linalool	Terpenoids	78-70-6	0.15 ± 0.00 d	0.64 ± 0.04 c	1.05 ± 0.04 b	1.31 ± 0.06 a
8	Indole	Heterocyclics	120-72-9	/	/	/	/
9	Methyl anthranilate	Ester	134-20-3	0.04 ± 0.00 d	0.22 ± 0.02 c	0.54 ± 0.05 b	0.91 ± 0.06 a
10	.alpha.-Farnesene	Terpenoids	502-61-4	/	/	/	/
11	3-Hexen-1-ol, benzoate, (Z)-	Ester	25152-85-6	/	/	/	/
12	JIF	/	/	/	/	/	/

## Data Availability

The original contributions presented in this study are included in the article/Supplementary Material. Further inquiries can be directed to the corresponding author.

## Supplementary Materials

The following supporting information can be downloaded at https://www.mdpi.com/article/10.3390/foods15193484/s1. Table S1. Identification and relative quantification of VOCs in CK, T1, T2 and T3; Table S2. Identification and relative quantification of VOCs in MCK, MT1, MT2 and MT3; Table S3. Eighteen unique compounds in light milk teas prepared from scented tea bases; Table S4. The differential compound with VIP ≥ 1, fold change > 2 or <0.5 and OAV ≥ 1 among T3 vs. T1, T2 vs. T1, T3 vs. T2, MT3 vs. MT1, MT2 vs. MT1 and MT3 vs. MT2.

## Abbreviations

The following abbreviations are used in this manuscript:

JTF	Jasmine tea flavor
TF	Transfer factor
RTD	Ready to drink
rOAV	Relative odor activity value
ANOVA	One-way analysis of variance
MS	Mass spectrometry
SPME	Solid phase microextraction
HS	Headspace
GC-MS	Gas chromatography-mass spectrometry
NIST	National institute of standards
RI	Retention indices
RT	Retention time
PCA	Principal component analysis
OPLS-DA	Orthogonal partial least squares-discriminant analysis
VIP	Variable importance in the projection
